# Diffusion Profiles of Health Beneficial Components from Goji Berry (*Lyceum barbarum*) Marinated in Alcohol and Their Antioxidant Capacities as Affected by Alcohol Concentration and Steeping Time

**DOI:** 10.3390/foods2010032

**Published:** 2013-01-25

**Authors:** Yang Song, Baojun Xu

**Affiliations:** 1 School of Life Sciences, The Chinese University of Hong Kong, Hong Kong; E-Mail: sunny9025@126.com; 2 Food Science and Technology Program, Beijing Normal University-Hong Kong Baptist University United International College, Zhuhai, Guangdong 519085, China

**Keywords:** goji berry, betaine, β-carotene, phenolics, antioxidant, steeping time, alcohol concentration

## Abstract

The fruit (goji berry) of *Lycium barbarum*, a traditional Chinese medicine, has been widely used in health diets due to its potential role in the prevention of chronic diseases. One of the most popular applications of goji berry is to make goji wine in China by steeping goji berry in grain liquor. However, how the steeping process affects antioxidant capacities and phytochemicals of goji berry is not yet fully understood. Therefore, to provide scientific data for the utilization of goji berry in the nutraceutical industry, the diffusion rate of betaine, β-carotene, phenolic compounds in goji berry and their antioxidant capacities affected by alcohol concentration and steeping time were determined by UV-Visible spectrophotometer. The results showed that low alcohol concentration (15% or 25%) would promote the diffusion of betaine and increase antioxidant activity, while high concentration (55% or 65%) would generally increase the diffusion of flavonoids and reduce antioxidant activity. The steeping time had no significant effect on the diffusion of phenolic compounds and antioxidant activities. However, all goji berry wine steeped for 14 days with different alcohol concentrations exhibited the highest betaine concentration. Current findings provide useful information for the nutraceutical industries to choose proper steeping time and alcohol concentration to yield desired health promotion components from goji.

## 1. Introduction

Goji berry species are deciduous woody perennial plants which are grown in the northwestern part of China, primarily in the Ningxia Hui Autonomous Region. Goji berry is prized for its versatility of color and nut-like taste in common meals, snacks, beverages, and medicinal applications. It has been widely used as a functional ingredient in nutraceuticals since excessive studies have been carried out and demonstrated that goji berry act as a crucial role in improving of vision, prevention of aging and age-related diseases, inhibition of cancer development and boosting immune system [1,2]. These healthy contributions have been proved and associated with the presence of various functional components including betaine, phenolics, β-carotenes and polysaccharides.

Beside the dry fruit of goji berry sold in the market, the most notable products are the goji beverage and goji wine (goji marinated in grain liquor) which have been recognized as functional drinks. Based on the principle of Chinese medicine, the grain liquor can act as organic solvent to enhance the medicinal function of herbal medicine and food materials due to the solubility of active compounds [3]. Therefore, the functional compounds of goji berry can be extracted by grain liquor.

Betaine, one of the most notable components in goji berry, known as trimethylglycine (TMG), is a metabolic product of choline. Betaine from goji berries helps to relieve anxiety, increases memory, promotes muscle growth and protects against fatty liver illness [4]. Many important biochemical processes rely on methylation, including the metabolism of lipids, neurotransmitters, and DNA [5]. It is well known that methylation ability of human body declines with age, therefore the decline of methylation contributes to the aging process. Betaine supplementation, therefore, has an interesting potential prevention benefit against the aging process [6].

Extensive researches have indicated that the antioxidant activity of goji berry is related to β-carotene and phenolic compounds [7,8]. The attractive red-yellow color of goji wine is mainly associated with a group of lipophilic compounds, known as carotenoids [9]. The carotenoids have been identified to be effective in preventing chronic diseases, such as cardiovascular diseases and skin cancer [10,11]. The β-carotene which is chemically a terpenoid accounts for the major portion of carotenoid in goji berry [12,13]. Other antioxidants existing naturally in goji berry are polyphenols, including tannins, lignans, flavonoids, and some other simple phenolic compounds. External stimuli (microbial infections, ultraviolet radiation, and chemical stressors) induce their synthesis to defense against herbivores and pathogens [14]. It is widely accepted that significant antioxidant activity of food is related to high total phenolics content [15]. Therefore, the use of flavonoid or other phenolics in diets and medicine has been intensively studied, particularly as treatments for neurodegenerative diseases and aging-related diseases [16]. Recent researches have strengthened the importance of flavonoids because of their imperative roles in antioxidant activities and other biological activities [17]. Several methods have been intensively employed to measure the antioxidant capacity, such as DPPH, or ferric reducing antioxidant power (FRAP).

This study is aimed at determining the content of these functional components and to assess the diffusion profiles of these components and investigate how antioxidant capacity influences by alcohol concentration and steeping time.

## 2. Materials and Methods

### 2.1. Food Materials and Chemicals

Ningxia goji berries were purchased from a local supermarket in Zhuhai, China. 2-Diphenyl-1-picryhydrazyl (DPPH), betaine, and 6-hydroxy-2,5,7,8-tetramethylchroman-2-carboxylic acid (Trolox) were purchased from Sigma-Aldrich Co. (Munich, Germany). Folin-Ciocalteu reagent was supplied by Sinopharm Chemical Reagent Co., Ltd. (Beijing, China). Catechin, Reinecke’s salt and triphenyltetrazolium chloride (TPTZ) were supplied by Yuanye Biotechnical Company (Shanghai, China). Gallic acid was purchased from Damao Chemical Reagent Company (Tianjin, China). Other chemicals were purchased from Guangzhou Chemical Reagent Company (Guangzhou, China). Unless otherwise stated, all the chemicals were of analytical grade.

### 2.2. Sample Preparation

Due to grain liquor in market can be categorized into three main groups, including low, medium, and high concentration alcohol. Therefore one concentration of alcohol was selected as low concentration group and different concentrations of alcohol were selected in both medial and high concentration groups. Therefore 30 treatments were obtained by combining five different concentrations (15%, 25%, 38%, 55%, and 65%) of alcohol and six different steeping times (1 day, 3 days, 5 days, 7 days, 10 days and 14 days). Each group was steeped in triplicate. Therefore total 90 samples were prepared. Goji berry (5 g) and 45 mL of alcohol solution were added into individual Erlenmeyer flask (the ratio of goji berry and alcohol is referred to the ratio of making commercial goji wine). The Erlenmeyer flasks were sealed with parafilm and tinfoil. The solid and liquid phases were separated by filtration with funnel and filter paper after finishing steeping process at room temperature. All liquid samples were stored in refrigerator at 40 °C for further analysis.

### 2.3. Determination of Betaine

According to the report of Zhu [6], trimethylglycine (betaine) can form aubergine color precipitate with Reineche’s salt after hydrolyzation. Then this color can be measured by UV-spectrophotometer and the results could be used for the calculation of betaine content.


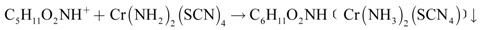


Therefore 20 mg of betaine standard weighed accurately and dissolved in 15.4 mL of distilled water to make 1.3 mg/mL stock standard solution. Then 0.8 mL, 1.6 mL, 2.4 mL, 3.2 mL, and 4 mL stock standard solution was transferred using pipettes to centrifugal tubes, respectively. The stock standard solutions were diluted to the 12 mL mark line with distilled water. Hydrochloric acid (2 M) was used to adjust the pH of standard solution to 1 and the samples were cooled down to 0–4 °C. After cooling, 8 mL of 3% Reinecke’s salt solution (made fresh) was added in each sample tube and the aging process of samples were carried out for 3–4 h in 0–4 °C. By using glass sand funnel, the aubergine color precipitate was filtered and washed by diethyl ether until the filtrate was colorless. Then the precipitates on filter paper were dissolved with 70% acetone solution and be transferred to 10 mL volumetric flask and diluted to mark line with distilled water. The absorbance of standard solution was measured and recorded using a UV-Visible spectrophotometer at 525 nm against 70% acetone solution as blank. All absorbance number was used to build up a standard curve correlated to known concentration of standard solution, which would be used to read and calculate the betaine content of samples.

Certain amount of sample (14 mL) of each tube was sucked into test tubes and the pH of samples was adjusted to 1 using 2 M hydrochloric acid. Then the samples were followed the procedures of standard solution to measure the absorbance of sample and to determine the betaine content in goji wine samples. 

### 2.4. Determination of β-Carotene

The quantitative analysis of β-carotene was conducted as the following procedures: 4 mL of goji wine sample was added in cuvette and then measured with the UV-Visible spectrophotometer at both 405 nm and 503 nm. The absorbance reading was used to calculate β-carotene content in sample according to the empirical equation [18] as below: C_β-carotene _= 4.642 × A_450_ − 3.091 × A_503_. Where C is the concentration of carotenoid expressed in μg/mL, and A450 and A503 represent the absorbance at 450 nm and 503 nm, respectively.

### 2.5. Determination of Phenolic Compounds

#### 2.5.1. Determination of Total Phenolic Content (TPC)

The TPC was determined by a Folin-Ciocalteu assay [19] with slight modifications [14] using gallic acid (GA) as the standard. The absorbance was measured with the UV-Visible spectrophotometer at 765 nm. The TPC was expressed as milligrams GA equivalents per gram goji berry (mg GAE/g) on a dry weight basis for solid sample and milligrams GA equivalents per milliliter goji wine (mg GAE/mL) for liquid sample through the calibration curve of GA. The linearity range of the calibration curve was 50–1000 μg/mL (*r* = 0.99). 

#### 2.5.2. Determination of Total Flavonoid Content (TFC)

The TFC was determined using a colorimetric method described previously [14]. The absorbance of standard solution, blank and samples were measured against the blank at 510 nm using the UV-Visible spectrophotometer. The TFCs were expressed as milligrams catechin equivalents per gram goji berry (mg CAE/g) on a dry weight basis or milligrams catechin equivalents per milliliter goji wine using the calibration curve of (+)-catechin. The linearity range of the calibration curve was 400–10000 μg/mL (*r* = 0.98).

### 2.6. Antioxidant Capacity Measurement

#### 2.6.1. *In Vitro* DPPH Free Radical Scavenging Capacity Analysis

The DPPH free radical scavenging capacity of goji wine was evaluated according to our previous communication [14]. The absorbance of all samples, blank, and standards were measured against the blank at 517 nm using the spectrophotometer. The scavenging rate was calculated as the equation: The scavenging rate (%) = (A_blank_ − A_sample_)/A_blank_ × 100% (A represent absorbance). The DPPH antioxidant values were also expressed as micromoles of Trolox equivalents per gram goji berry (μmol TE/g) on a dry weight basis using the calibration curve of Trolox. The linearity range of the calibration cure was 20–1000 μM (*r* = 0.99). 

#### 2.6.2. The Ferric Reducing Antioxidant Power (FRAP) Assay

The FRAP was performed as described previously [14,20]. The absorbance of all samples, blank, and standard solutions were measured against the blank at 593 nm by the spectrophotometer under condition of 37 °C water bath. The FRAP value was expressed as millimoles of Fe^2+ ^equivalent (FE) per gram goji berry (mmol FE/g) on a dry weight basis using the calibration curve of Fe^2+^. The linearity range of the calibration curve was 0.1–1.0 mM (*r* = 0.99).

### 2.7. Statistical Analysis

All steeping treatments were carried out in triplicates. Analyses were done based on triplicate samples. Means, standard deviations were done using Microsoft Excel 2003. Significant differences of the results for part of the experiment were statistically analyzed by SPSS 14.0. Statistical significance was accepted at a level of *p* < 0.05.

## 3. Results and Discussion

### 3.1. Betaine Content in Goji Wine

**Figure 1:**
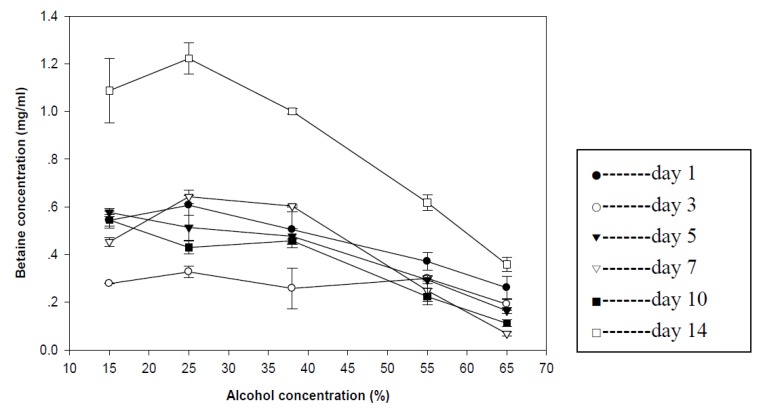
Betaine concentration in goji wine. Results were expressed as mean ± standard deviation (*n* = 3).

The betaine concentration of goji wine varied at the range from 0.68 mg/mL to 0.07 mg/mL with different treatments (Figure 1). The goji wine with high alcohol concentration presented the low betaine concentration and the goji wine with 25% of alcohol concentration presented the best diffusion efficacy of betaine diffusion. 

The goji wine treated with 25% alcohol and steeped for 14 days presented the highest betaine content (8.94 mg/g dry basis). The result also indicated that the commercial goji wine only had 2.13 mg/g betaine diffused from goji berry. That might be due to the fact the diffusion profile of betaine content in goji wine was fluctuated. Therefore, manufacturer could separate the goji berry from goji wine timely to preserve the functional components as much as possible. Two weeks of steeping time with 25% of alcohol may be one of the best processing conditions. 

### 3.2. β-Carotene Content in Goji Wine

**Figure 2:**
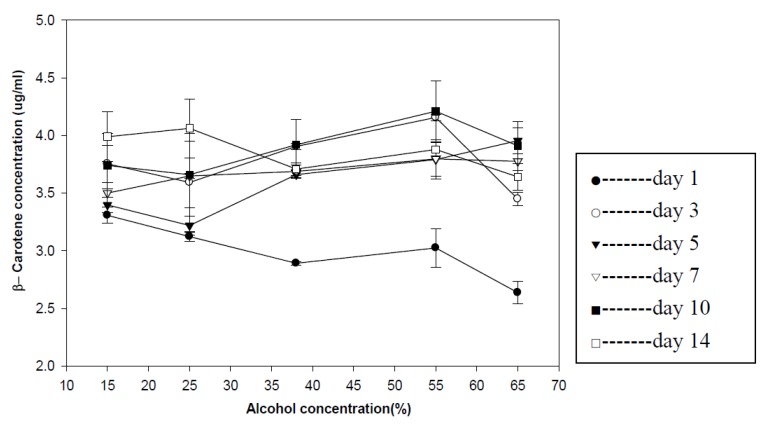
β-Carotene concentration in goji wine. Results were expressed as mean ± standard deviation (*n* = 3).

The β-carotene concentration of goji wine with different treatments was in the range of 2.64 μg/mL to 4.21 μg/mL (Figure 2). The goji wine steeped for one day had the lowest concentration of β-carotene and other goji wines had the similar concentration of β-carotene. The results showed that the different alcohol concentration had no significant effect on the diffusion of β-carotene in goji wine. 

The diffusion of β-carotene in goji wine was influenced by the steeping time, but not alcohol concentration. In addition, the β-carotene content in dry goji berry was 82.2 μg/g based on the research of Li [21]. Therefore, all the results in the experiment also had proven the high extraction efficacy of alcohol on β-carotene.

### 3.3. Phenolic Compound Content in Goji Wine

#### 3.3.1. Total Phenolic Content in Goji Wine

**Figure 3:**
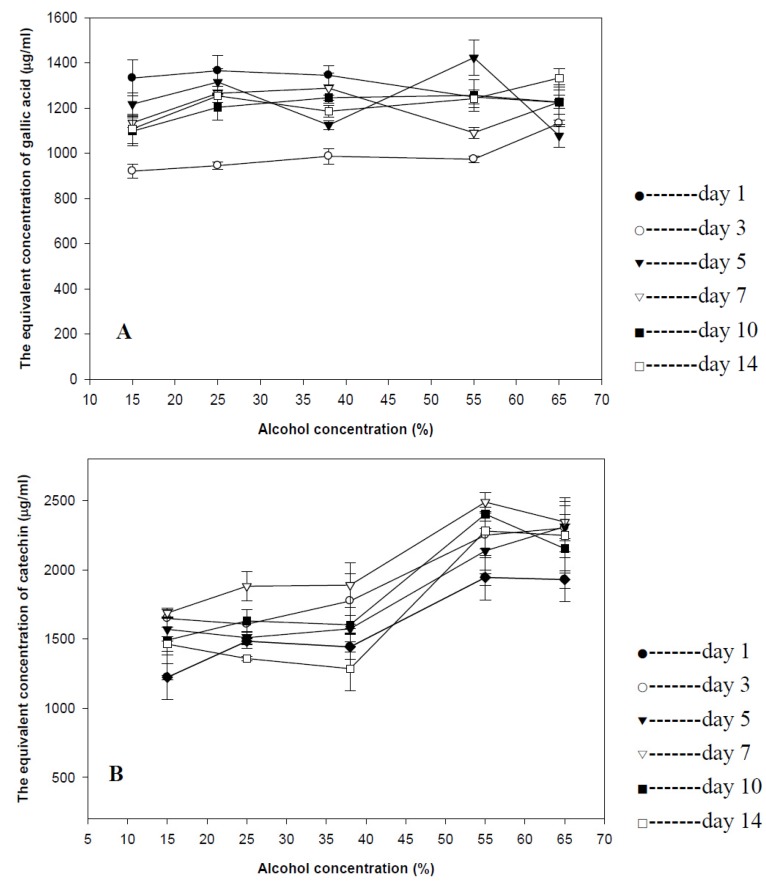
Phenolic content of goji wine. (**A**) Total phenolic content of goji wine; (**B**) total flavonoid content of goji wine. Results were expressed as mean ± standard deviation (*n* = 3).

The equivalent concentration of gallic acid of goji wines with different treatments were plotted in Figure 3A. The figure indicated that all goji wines had similar equivalent concentration of gallic acid in range of values between 1.08 mg/mL to 1.42 mg/mL except for the the goji wines steeped for 3 days (0.99–1.13 mg /mL), the results of which were significantly lower than any other goji wines. Therefore, the result may be concluded that the TPC was neither dependent upon the concentraiton of alcohol nor the steeping time.

#### 3.3.2. Total Flavonoid Content in Goji Wine

The equivalent concentration of catechin of goji wines with different treatments was plotted in Figure 3B. The figure demonstrated that the steeping time had no significant effect on diffusion of total flavonoid in goji wine, but the alcohol concentration played the predominant role in the diffusion of total flavonoid in goji wine. To be more specific, the high alcohol concentration would promote the diffusion of total flavonoid (1.93–2.49 mg/mL) compared with the low alcohol concentration (1.22–1.89 mg/mL).

### 3.4. Antioxidant Capacity of Goji Wine

#### 3.4.1. DPPH Free Radical Scavenging Capacity of Goji Wine

**Figure 4:**
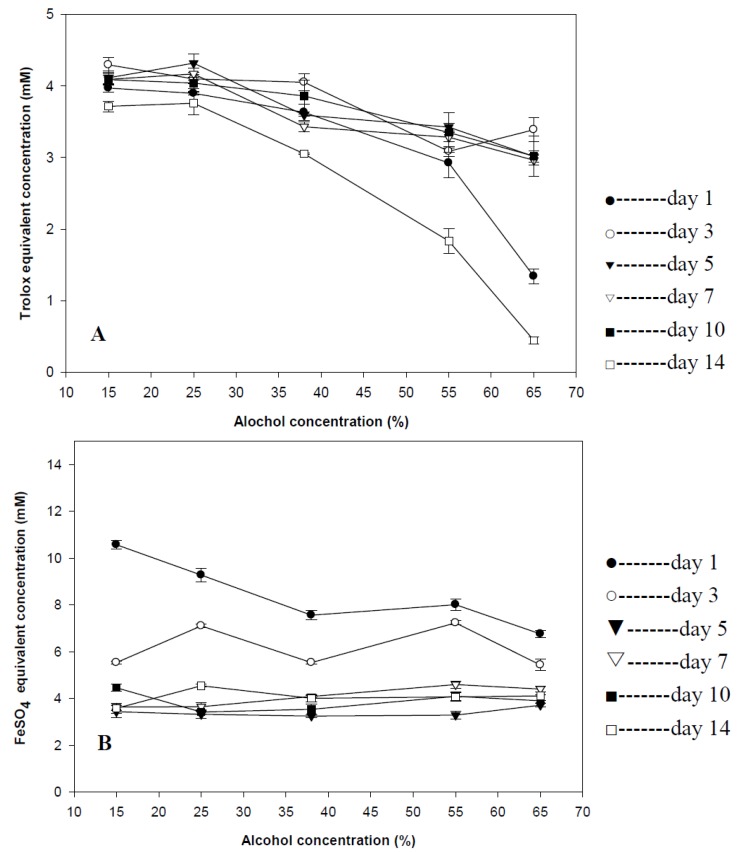
Antioxidant activities of goji wines. (**A**) 2-Diphenyl-1-picryhydrazyl (DPPH) free radical scavenging capacities of goji wine; (**B**) ferric reducing antioxidant power of goji wine. Results were expressed as mean ± standard deviation (*n* = 3).

The antioxidant capacity of goji wine was affected by both the steeping time and alcohol concentration based on DPPH assay (Figure 4A). To be more specific, the antioxidant capacity was relative stronger with the shorter steeping time and lower alcohol concentration (4.32 mM) compared with other goji wine with longer steeping time or high alcohol concentration (0.45 mM). The result also indicated that the commercial goji wine had 4.19 mM of Trolox equivalent concentration which was almost the highest antioxidant capacity of goji wine in this investigation.

#### 3.4.2. Ferric Reducing Antioxidant Power of Goji Wine

Another method of the antioxidant capacity measurement also confirmed that goji wines with shorter steeping time had the stronger antioxidant capacity (Figure 4B). For example, the ferrous sulfate equivalent concentration of goji wine treated with 15% alcohol and steeped for 1 day was 10.57 mM. The alcohol concentration, however, was not a critical factor affecting the antioxidant capacity of goji wine.

The samples with high DPPH scavenging capacity (≥30 mmol Trolox equivalents/g) were selected and arranged in order. The results revealed that these functional components did not exhibit a strong proportional correlation. The goji wine with high TPC, TFC or β-carotene content just has moderate or low antioxidant activity but some goji wine with medium TPC, TFC or β-carotene content have strong antioxiant activity. Overall, the steeping time has no significant effect on diffusion of total flavonoid in goji wine but the alcohol concentration plays the predominant role in influencing the diffusion of total flavonoid in goji wine.

## 4. Conclusions

Goji wines have already widely accepted by most elder people and are recognized as one of the functional wine against aging and aging-related disease. Nevertheless, the concentration of alcohol added and the steeping time are not explicated by most manufacturers. The present investigation shows that low alcohol concentration can promote the diffusion of functional components in large extent, except the total flavonoid content which is boosted by high alcohol concentration. However, the steeping time has no significant effect on the diffusion of functional components and basically the diffusion profile is fluctuated during the soaking process. The antioxidant activity may influenced by steeping time and combined action of β-carotene, TPC, TFC, and other active compounds. The high content of β-carotene, TPC and TFC can develop moderate antioxidant capacity. Therefore, some indications could be summarized for goji wine industries. Firstly, the manufacturers could treat goji berry with 25% of alcohol and separate the goji berry from goji wine after 14 days to obtain goji wine containing high betaine. Secondly, the goji berry should be treated with low concentration alcohol and relative short marinating time (less than 7 days) to obtain high betaine and antioxidant components. Thirdly, if the target customers demand vision beneficial goji wine, manufactures could employ 55% of alcohol and soak for at least 14 days to gain high β-carotene content.
